# Age increases MCP‐1 level in association with bariatric surgery operating time and metabolic risk severity

**DOI:** 10.1002/osp4.105

**Published:** 2017-04-26

**Authors:** S. K. Malin, J. L. Kaplan, L. Meng, J. C. Garmey, J. L. Kirby, A. M. Taylor, P. T. Hallowell, C. A. McNamara

**Affiliations:** ^1^ Department of Kinesiology University of Virginia Charlottesville USA; ^2^ Divison of Endocrinology and Metabolism University of Virginia Charlottesville USA; ^3^ Robert M. Berne Cardiovascular Research Center University of Virginia Charlottesville USA; ^4^ Department of Biochemistry, Molecular Biology, and Genetics University of Virginia Charlottesville USA; ^5^ Department of Surgery University of Virginia Charlottesville USA; ^6^ Division of Cardiovascular Medicine University of Virginia Charlottesville USA; ^7^ Beirne B. Carter Center for Immunology Research University of Virginia Charlottesville USA; ^8^ Department of Molecular Physiology and Biological Physics University of Virginia Charlottesville USA

**Keywords:** Glycaemic control, inflammation, insulin resistance, metabolic syndrome

## Abstract

**Objective:**

Assess the role of inflammation on operating time in younger vs. older bariatric surgery patients.

**Methods:**

Fifty‐five younger (F: 46, Age: 34.9 ± 4.0 years, body mass index [BMI]: 48.2 ± 1.0 kg m^−2^) and 48 older (F: 34, Age: 57.0 ± 5.1 years, BMI: 46.8 ± 1.0 kg m^−2^) adults were studied prior to surgery. Blood pressure, glycaemic control (fasting glucose/insulin, HbA_1c_), lipids (high‐density lipoprotein and triglycerides) and inflammation (monocyte chemoattractant protein‐1 [MCP‐1]) were assessed. Metabolic risk severity *z*‐scores were calculated from clinical outcomes. Omental adipose biopsies were collected at surgery for MCP‐1 protein analysis. Operating time was used to characterize surgical difficulty.

**Results:**

Older vs. younger adults had higher HbA_1c_ (*P* = 0.03). There was no difference in BMI, lipids, metabolic risk severity or insulin between groups, but operating time was longer in older vs. younger individuals (*P* = 0.04). Circulating MCP‐1 was also elevated in older vs. younger adults (*P* = 0.04) independent of HbA_1c_, although this was not explained by omental fat. Nevertheless, serum MCP‐1 was associated with increased metabolic risk severity (*R* = 0.27, *P* = 0.01). In addition, operating time was linked to HbA_1c_ (*R* = 0.30, *P* = 0.01) and omental MCP‐1 protein (*R* = 0.31, *P* < 0.01).

**Conclusions:**

MCP‐1 is associated with longer operating time and increased metabolic risk severity in older bariatric patients independent of glycaemic control. Pre‐operative treatment of inflammation may be required to enhance surgery effectiveness.

## Introduction

Bariatric surgery has emerged as an effective therapy for combating the rise in obesity and associated metabolic abnormalities (e.g. hyperglycaemia, inflammation and insulin resistance) 1. However, there is controversy on the implementation of bariatric surgery across the age range 2. In fact, few data exist examining the risks and/or benefits of bariatric surgery in older adults 3, 4, 5, 6, 7, 8, 9. Interestingly, some 10, 11, 12 but not all 3, 7, 9 studies report that older people have smaller weight loss and comorbidity resolution following surgery. In addition, there appears to be increased incidents of longer lengths of stay, surgical complications and increased mortality risk 30 days post‐operation in the older population 5, 11, 13, 14, 15. An important knowledge gap in the literature though is an explanation for how ageing contributes to bariatric surgery difficulty.

The mechanism by which ageing contributes to surgical difficulty is likely multifactorial, but a leading candidate is related to excess body fat, which contributes to adiposopathy, or ‘sick fat’ that in turn induces insulin resistance and β‐cell dysfunction 16. Indeed, monocyte chemoattractant protein‐1 (MCP‐1) is a chemokine linked to chronic low‐grade inflammation and macrophage infiltration in adipose tissue 17. Macrophage infiltration in adipose is important because oxidative stress and inflammatory cytokines exacerbate insulin resistance and promotes dysregulation of glucose as well as increases risk of atherosclerosis 18, 19, 20. Collectively, this heightened metabolic risk severity could increase risk for surgical complications and/or make surgery more difficult. Circulating MCP‐1 is higher in obese 21 and type 2 diabetic patients 22, and visceral adipose tissue has higher levels of MCP‐1 compared with other depots 23. This later observation is consistent with the view that increased visceral adiposity is associated with metabolic syndrome severity and insulin resistance 24. Together, these findings suggest that targeting reductions in abdominal fat inflammation may reduce surgical difficulty, complication rates and operating time 25, 26. Thus, understanding the role of MCP‐1 on surgical difficulty may have clinical and public health relevance for identification of metabolic profiles in ageing individuals that lead to improved patient care. However, to date, no study has determined whether age impacts operating time in relation to MCP‐1. Moreover, few data have stratified age by diabetes status to further characterize the relationship between MCP‐1 and operating time. Therefore, it was hypothesized that older individuals would be characterized by elevated MCP‐1 compared with younger adults, and this less favourable MCP‐1 profile (e.g. blood and adipose tissue) would correlate with bariatric surgery operating time and metabolic risk severity independent of glycaemic control (i.e. HbA_1c_).

## Methods

### Subjects

This was a retrospective analysis of a cross‐sectional study of younger (i.e. <40 years) and older (i.e. >50 years) adults undergoing bariatric surgery (e.g. Roux‐en‐Y gastric bypass, sleeve gastrectomy or gastric banding; Table 1). A subset of 103 out of 196 patients was ranked based on age, and only younger and older adults were included to test if age impacts inflammation in relation to operating time. Individuals between the ages of 41 and 49 years were excluded to assess the effects of ageing on outcomes of interest. Prior to study enrolment, our nutrition, psychology, bariatricians and surgery teams cleared subjects for bariatric surgery by use of medical examinations that included resting electrocardiogram, urinalysis and blood biochemistry. Operating time was defined as time of incision to time of close and used to characterize surgical difficulty, and length of stay post‐operation and 30 day readmission rates were also assessed. Participants were verbally briefed about the study and signed informed consent documents approved by the University of Virginia Institutional Review Board.

**Table 1:** Comparison of pre‐operative demographics between young and old subjects.

	Young pooled	Old pooled	*P*‐value	Young‐NGT	Young‐T2D	Old‐NGT	Old‐T2D	*P*‐value#
Population (*n,* M/F)	55, 10/45	48, 14/34	0.24	40, 7/33	15, 3/12	14, 5/9	34, 9/24	**—**
Age (y)	34.9 ± 4.0	57.0 ± 5.1	<0.001	34.5 ± 0.6	35.9 ± 0.7	55.7 ± 1.2*, ^	57.5 ± 0.8 *, ^	<0.001
Type 2 diabetes (*n*, %)	15, 27%	25, 52%	0.01	**—**	**—**	**—**	**—**	**—**
Hypertension (*n*, %)	27, 49%	38, 79%	0.002	19, 47%	8, 53%	9, 64%	29, 85%	**—**
Metabolic syndrome (*n*, %)	44, 80%	45, 94%	0.04	29, 72%	15, 100%	12, 85%	33, 97%	**—**
Metabolic risk severity (*z*‐score)	3.1 ± 0.3	3.4 ± 0.4	0.65	2.3 ± 0.3	5.0 ± 0.8*	1.3 ± 0.4^	4.3 ± 0.5*, †	<0.001
Medications								
Insulin or insulin secretagogue (*n*, %)	8, 14%	20, 42%	0.003	0, 0%	8%	0, 0%	20, 58%	**—**
Beta‐blockers (*n*, %)	7, 13%	19, 40%	0.003	6, 15%	1, 6%	2, 14%	17, 50%	**—**
NSAIDS (*n*, %)	7, 13%	12, 25%	0.06	5, 12%	2, 13%	6, 42%	6, 17%	**—**
Oral contraceptives (*n*, %)	2, 4%	0, 0%	0.49	2, 5%	0, 0%	0, 0%	0, 0%	**—**
Surgery type								
RYGB (*n*, %)	29, 52%	23, 47%	0.28	20, 50%	9, 60%	5, 35%	21, 61%	**—**
SG (*n*, %)	7, 12%	6, 12%	1.00	5, 12%	2, 13%	3, 21%	10, 29%	**—**
Gastric banding (*n*, %)	19, 34%	16, 33%	0.66	15, 37%	4, 26%	6, 42%	3, 8%	**—**
Anthropometrics								
Weight (kg)	138.7 ± 30.6	131.1 ± 22.0	0.16	134.0 ± 4.3	150.8 ± 9.5	124.3 ± 4.1	133.8 ± 4.1	0.06
BMI (kg m^−2^)	48.2 ± 1.0	46.8 ± 1.0	0.34	47.1 ± 1.0	50.8 ± 2.3	44.6 ± 1.1	47.7 ± 1.3	0.14
Glucose metabolism								
Fasting glucose (mg dL^−1^)	120.2 ± 5.0	135.3 ± 7.3	0.09	108.6 ± 4.6	150.7 ± 10.7*	96.8 ± 4.5	151.1 ± 8.9*, †	<0.001
Fasting insulin (μU mL^−1^)	16.9 ± 3.5	18.8 ± 4.1	0.72	11.7 ± 1.5	30.5 ± 11.9*	7.4 ± 0.8^	23.5 ± 5.7*, †	<0.001
HbA_1C_ (%)	6.3 ± 1.3	7.1 ± 1.5	0.03	5.6 ± 0.1	7.2 ± 0.5*	5.8 ± 0.1	7.3 ± 0.3*	<0.001
HOMA‐IR	6.0 ± 1.6	7.2 ± 2.0	0.62	2.9 ± 0.4	8.8 ± 3.1	2.2 ± 0.3	9.6 ± 3.0	0.02
Lipids and blood pressure								
HDL (mg dL−1)	39.9 ± 1.5	40.4 ± 1.3	0.83	41.3 ± 1.8	36.0 ± 2.4	40.6 ± 2.4	40.2 ± 1.7	0.89
Triglyceride (mg dL^−1^)	149.1 ± 10.0	162.8 ± 11.0	0.36	145.3 ± 10.9	158.8 ± 23.6	144.2 ± 13.1	170.3 ± 14.6	0.51
Systolic blood pressure (mmHg)	131.3 ± 3.2	136.1 ± 3.2	0.22	131.4 ± 2.4	130.8 ± 6.1	128.0 ± 6.5	139.4 ± 3.6	0.19
Diastolic blood pressure (mmHg)	73.3 ± 1.5	72.8 ± 1.8	0.84	74.2 ± 1.7	70.8 ± 3.2	72.2 ± 4.4	73.1 ± 2.0	0.82
Pulse pressure (mmHg)	57.9 ± 1.8	63.2 ± 2.2	0.07	57.3 ± 1.9	60.4 ± 4.6	55.7 ± 3.5	66.4 ± 2.8*	0.03
Mean arterial pressure (mmHg)	92.6 ± 1.6	93.9 ± 2.1	0.63	93.3 ± 1.7	90.8 ± 3.8	90.8 ± 4.9	95.2 ± 2.3	0.65

Data are reported as mean ± standard error of the mean or count/percentage when appropriate.

# *P*‐value represents analysis by anova.

* Compared with Young (*P* < 0.05).

^ Compared with Young + T2D (*P* < 0.05).

† Compared with Old (*P* < 0.05).

BMI, body mass index; HDL, high‐density lipoprotein; HOMA‐IR, homeostasis model assessment of insulin resistance; NSAIDS, non‐steroidal anti‐inflammatory drugs; Normal glucose tolerant (NGT); Type 2 diabetes (T2D); RYGB, Roux‐en‐Y gastric bypass; SG, sleeve gastrectomy; T2D, type 2 diabetes.

### Anthropometrics and blood pressure

Subjects reported to the Department of Surgery for screening purposes prior to surgery. Height and weight were obtained in a standard hospital gown on a calibrated scale and wall‐mounted stadiometer. Body mass index (BMI) was calculated as body mass (kg) divided by height (m)^2^ to characterize obesity. Research nurses used an automated platform (DINAMAP™ Procare 400, GE Medical Systems, Milwaukee, WI) to obtain morning systolic (SBP) and diastolic (DBP) measures, which was performed on the left arm in a low‐light room while participants lay semisupine after 10 min of awake rest. Reported data are based on the average of three measurements, with 1 min between each measure. Mean arterial pressure (MAP) was calculated as MAP = 2/3(DBP) + 1/3(SBP). Pulse pressure was estimated by subtracting DBP from SBP.

### Clinical labs

After an approximate 10‐h overnight fast, an indwelling catheter was placed in an antecubital vein for collection of blood samples. MCP‐1, glucose, lipids (i.e. high‐density lipoprotein and triglycerides) and insulin were collected. Homeostasis model assessment of insulin resistance was calculated as fasting glucose (mM) × fasting insulin (μU mL^−1^) divided by 22.5 to estimate insulin resistance. Sex‐specific *z*‐scores were calculated to determine the effects of age on the severity of metabolic syndrome. Metabolic risk severity *z*‐scores were calculated from clinical cut points of high‐density lipoprotein (HDL), triglycerides (TG), fasting glucose (FPG), BMI, MAP and insulin using sex‐specific criteria: women: [(50‐HDL)/9.74] + [(TG‐150/75.58)] + [(FPG‐100)/44.8] + [(BMI‐30)/7.23] + [(MAP‐100)/13.2] + [(INSULIN‐20)/27.4]. Men: [(40‐HDL)/12.23] + [(TG‐150/75.58)] + [(FPG‐100)/44.88] + [(BMI‐30)/7.23] + [(MAP‐100)/13.2] + [(INSULIN‐20)/27.4] and were modified from prior work 24. National Cholesterol Education Program Adult Treatment Panel III criteria for metabolic syndrome were also calculated based on the sum of risk factors for metabolic syndrome.

### Biochemical analysis

Whole‐blood glucose was measured immediately after collection using the glucose oxidase method (YSI 2300 STAT Plus, Yellow Springs, OH). HbA_1c_ was measured in whole blood by capillary electrophoresis via the Sebia CAPILLARYS 2 Flex‐Piercing instrument (University of Virginia Laboratories, Charlottesville, VA). The remaining blood was centrifuged at 4°C for 10 min and frozen at −80°C until subsequent analysis. To minimize inter‐assay variability, blood measurements for each subject were analysed on the same plate. Plasma triglycerides and cholesterol were analysed using enzymatic methods with an automated platform (Roche Modular Diagnostics, Indianapolis, IN). Plasma insulin was assayed by RIA (Millipore, St. Charles, MO). MCP‐1 was assayed by ELISA (SABiosciences, Valencia, CA).

### Adipose biopsy and analysis

Omental and subcutaneous adipose biopsy samples were collected at the time of bariatric surgery and performed by the same surgeon (P. T. H.) to minimize variance in tissue collection. Human omental and subcutaneous adipose tissue was processed using published methods 27. Adipose tissue was homogenized in 2 mL RIPA buffer containing protease inhibitors and incubated on ice for 30 min. Protein lysate was collected and used for ELISA analysis. Human MCP‐1 (SABiosciences, Valencia, CA) levels were determined with commercial kits. Total protein concentration was determined with the Pierce BSA Protein Assay.

### Statistical analysis

Data were analysed using the statistical program R (Leopard build 64‐bit, Vienna, Austria 2013). Categorical group variables were assessed using Fisher exact tests. Two‐tailed paired *t*‐tests were used to analyse differences between younger and older adults. Because older adults had higher HbA_1c_ compared with younger adults, we co‐varied for glycaemic control to isolate the effects of age in relation to serum MCP‐1 and operating time. In addition, younger and older adults with and without diabetes were compared by anova to stratify clinical characteristics, and Bonferroni *post hoc* analysis was conducted to determine group differences. Pearson's product moment correlations were used to examine associations between outcomes. Data are expressed as mean ± standard error of the mean and significance was accepted as *P* ≤ 0.05.

## Results

### Demographics

**Figure 1:**
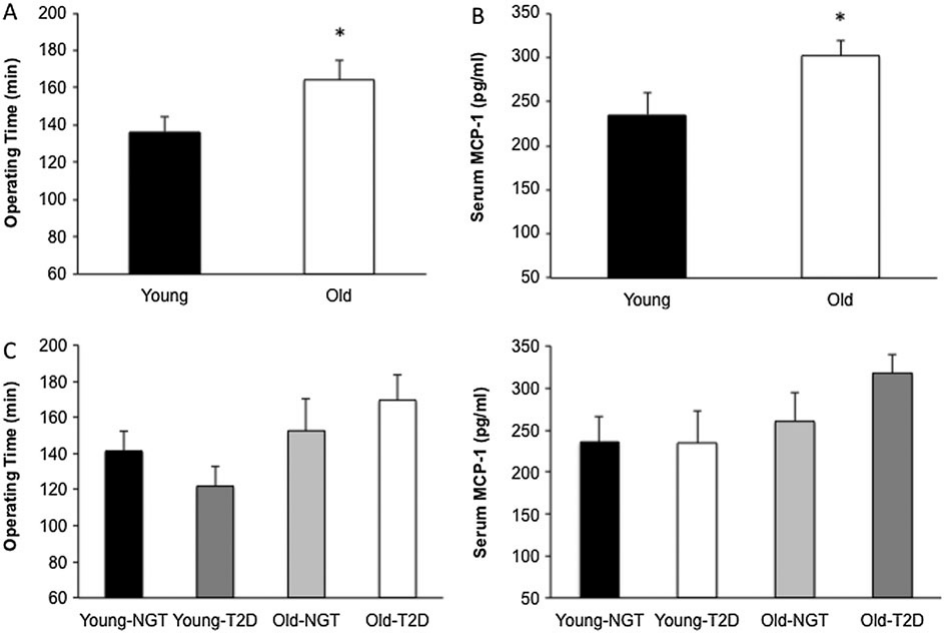
Relationship between age, operating time (a,c) and monocyte chemoattractant protein‐1 (MCP‐1) (b,d). Normal glucose tolerant = NGT. Type 2 diabetes = T2D. Data are mean ± standard error of the mean. *P = 0.04.

Older individuals had a higher prevalence of type 2 diabetes, hypertension and metabolic syndrome (all; *P* < 0.05), although metabolic risk severity was comparable with younger adults (Table 1). Older adults required higher medication usage predominantly from insulin (42 vs. 14%, *P* < 0.03) and beta‐blockers (40 vs. 13%, *P* < 0.03) but not non‐steroidal anti‐inflammatory drugs or oral contraceptives when compared with younger subjects. Although there were no differences in surgery type, length of stay (1.96 ± 0.38 vs. 2.1 ± 0.44 days, *P* = 0.81*)* or 30 day readmission rates post‐operation (4 vs. 6, *P* = 0.50) between younger and older groups, operating time was approximately 30 min longer in older compared with younger adults (*P* = 0.04; Figure 1a). Moreover, stratification of age based on type 2 diabetes (T2D) and normal glucose tolerant (NGT) status did not alter relationships with length of stay (Young‐NGT: 2.0 ± 0.5 vs. Young‐T2D: 1.8 ± 0.3 vs. Old‐NGT: 1.2 ± 0.2 vs. Old‐T2D: 2.4 ± 0.6 days, *P* = 0.65) or readmission rates (Young‐NGT: 3 vs. Young‐T2D: 1 vs. Old‐NGT: 2 vs. Old‐T2D: 4).

### Body weight and clinical labs

There were no statistical differences in body mass, insulin resistance, blood lipids or hypertension between younger and older adults (Table 1). However, HbA_1c_ was significantly higher in older compared with younger adults (*P* = 0.03, Table 1). As expected by study design, individuals with T2D had higher glucose and insulin levels compared with NGT individuals (Table 1).

### Circulating and adipose tissue monocyte chemoattractant protein‐1

**Figure 2:**
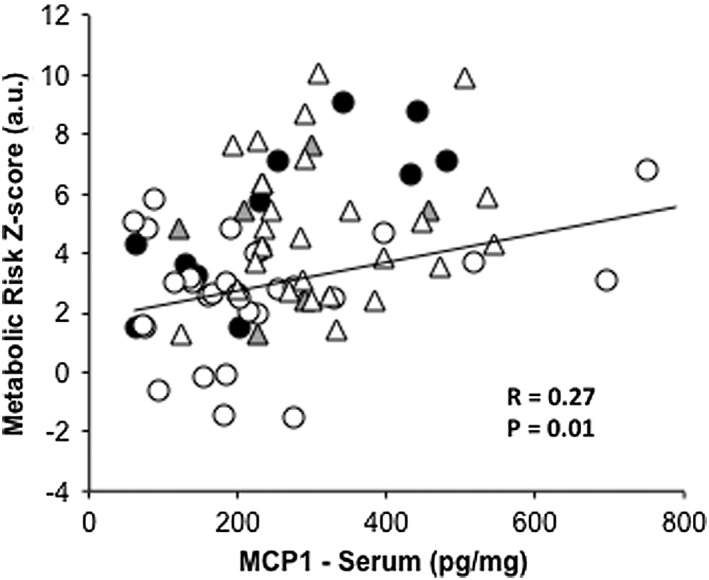
Correlation between inflammation and metabolic risk severity. Open circles, Young‐normal glucose tolerant (NGT); closed circles, Young‐type 2 diabetes (T2D); open triangles, Old‐NGT; closed triangles, Old‐T2D.

**Figure 3:**
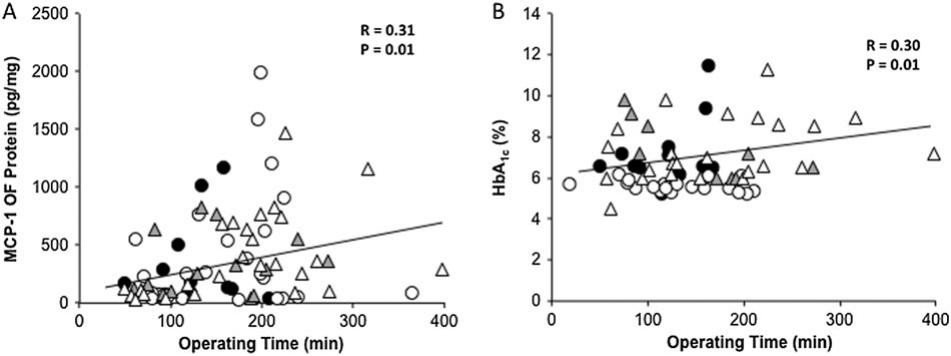
Correlation between operating time and monocyte chemoattractant protein‐1 (MCP‐1) OF Protein (a) and HbA_1c_ (b). Open circles, Young‐normal glucose tolerant (NGT); closed circles, Young‐type 2 diabetes (T2D); open triangles, Old‐NGT; closed triangles, Old‐T2D.

Serum MCP‐1 was statistically different between younger and older adults (*P* = 0.04; Figure 1b), and this remained significantly higher after adjusting for HbA_1c_. However, there was no statistical difference in MCP‐1 protein concentrations in omental (345.6 ± 58.1 vs. 353.6 ± 69.5 pg mg^−1^ total protein, *P* = 0.93) or subcutaneous adipose tissue (174.8 ± 53.0 vs. 123.4 ± 21.7 pg mg^−1^ total protein, *P =* 0.35) between older and younger adults. There was no statistical difference in MCP‐1 omental (Young‐NGT: 365.6 ± 89.0 vs. Young‐T2D: 323.6 ± 110.6 vs. Old‐NGT: 178.6 ± 70.8 vs. Old‐T2D: 395.0 ± 71.3 pg mg^−1^, *P* = 0.58) or subcutaneous adipose concentrations (Young‐NGT: 135.4 ± 27.1 vs. Young‐T2D: 74.8 ± 6.0 vs. Old‐NGT: 64.7 ± 14.1 vs. Old‐T2D: 229.7 ± 73.3 pg mg^−1^, *P* = 0.58) based on diabetes status. Nevertheless, MCP‐1 serum was significantly correlated with increased metabolic risk severity (*R* = 0.27, *P* = 0.01, Figure 2) but not BMI (*R* = 0.14, *P* = 0.14). MCP‐1 protein concentration in omental fat was significantly correlated with operating time (*R* = 0.31, *P* = 0.01; Figure 3). Operating time was also significantly associated with HbA_1c_ (*R* = 0.30; *P* = 0.01) and length of stay (*R* = 0.55; *P* < 0.001).

## Discussion

The major finding from this study was that older adults have longer operating times when compared with younger individuals, and this increased operating time was directly correlated to MCP‐1 and metabolic risk severity independent of glycaemic control (Figure 1). These findings are consistent with prior work reporting that fasting pre‐operative hyperglycaemia, inflammation, as well as long duration of diabetes 28, 29 predict smaller weight loss 30, reduced cardiorespiratory fitness 31, 32 and poor HbA_1c_ levels resolution following bariatric surgery 33. Interestingly, it was recently reported that obesity related metabolic risk severity at the time of surgery was directly related to reduced diabetes remission 10 years post‐gastric bypass surgery 34. Thus, these data add to this prior work by showing that age and MCP‐1 are clinical determinants of operating time, thereby potentially contributing to higher intra‐operative and post‐operative risk 35, 36.

The exact reason why ageing increases operating time is unclear, but it may relate to adiposity 16. Excess adiposity is known to contribute to surgical difficulty. If patients in our study had greater BMI than younger adults, it would reason that surgical difficulty is higher. However, BMI was comparable between groups and there was no significant correlation between BMI and serum MCP‐1, suggesting that obesity was not a factor driving differences in operating time or MCP‐1. It is worth noting though that we did not determine total (e.g. DEXA or BodPod) or visceral adiposity (e.g. magnetic resonance imaging or computerized tomography scans), and it remains possible that excess abdominal obesity contributed to surgical difficulty in older adults. Another possible reason for increased operating time in older adults may relate to adiposopathy 16. MCP‐1 is an inflammatory hormone demonstrated to increase atherosclerosis and decrease insulin sensitivity 17. In the current study, circulating MCP‐1 was elevated in older compared with younger adults, and was directly related to increased metabolic risk severity (Figure 2). In addition, the expression of MCP‐1 from omental fat was correlated with operating time (Figure 3). Together these findings suggest that inflammation may drive metabolic risk and complicate surgical procedures. Indeed, the relationships observed with serum MCP‐1 and metabolic risk severity (Figure 3) are consistent with prior work demonstrating that bariatric surgery‐induced weight loss decreased MCP‐1 concentrations 37 and macrophage infiltration 38. Moreover, these findings of elevated circulating MCP‐1 prior to bariatric surgery are consistent with prior work characterizing individuals with T2D non‐remission as having ongoing inflammation 17.

In an effort to understand the mechanism by which MCP‐1 was elevated in older adults and contributed to increased operating times, subcutaneous and omental fat was collected. Prolonged operating time was directly correlated with increased MCP‐1 protein concentration in omental but not subcutaneous adipose tissue (Figure 3). This association suggests that MCP‐1 secreted from omental fat depots is related to increased surgical difficulty 25, 26. How MCP‐1 secreted from visceral adiposity promotes increased operating time is beyond the scope of this study, but MCP‐1 is established to promote insulin resistance and increase risk for atherosclerosis 16, 39. To combat this pro‐inflammatory adipose tissue depot, very low calorie diet interventions ranging from 2 to 4 weeks are often advised to patients pre‐operatively to shrink liver size and central adiposity 25, 26. It is also important to recognize that a lack of aerobic fitness may have played a role in explaining the present relationships between adipose derived‐inflammation and operating time 31, 32. In either case, a somewhat surprising but not unexpected observation was that MCP‐1 protein concentrations were similar between older and younger bariatric patients in our study regardless of the adipose depot studied. This finding suggests that other tissues including macrophages, liver and/or skeletal muscle may have contributed to the circulating differences in MCP‐1. Thus, further work is needed to elucidate the mechanism by which inflammation contributes to operating surgery difficulty.

Previous work reports that poor glycaemic control characterizes individuals with a low propensity for T2D remission following bariatric surgery 30, 40. Interestingly, these findings are consistent with lifestyle intervention, whereby patients with chronic hyperglycaemia have blunted gains in aerobic fitness 41 as well as insulin sensitivity and fat oxidation 42. Indeed, recent work also demonstrates that bariatric surgery promotes better glycaemic control in individuals with short‐duration vs. long‐duration diabetes 2 years after bariatric surgery because of insulin secretion 28 and/or insulin sensitivity 43. Although age was observed to have an independent relationship with operating time, our current work suggests that individuals with higher circulating glucose concentrations had increased operating times, which in turn, was associated with prolonged hospital stays. These observations are consistent with views that early improvements in hyperglycaemia in bariatric patients may be needed to alleviate diabetes complications that increase risk for stroke, myocardial infarction, death and lower surgical risk 44.

This study has certain limitations that may affect interpretation of the results. It is recognized that the associations observed herein do not equate to causality and further prospective work is needed to determine if targeting adipose tissue inflammation produces improved surgical‐related outcomes during and following bariatric surgery. Given the nature of the present study design, we are not able to comment on liver size to determine operating difficulty. Nevertheless, a strength of this study is that the same surgeon performed all operations in this analysis, thereby minimizing concern of personnel differences between surgical types and/or groups. Another issue was that medications were avoided 24‐h prior to blood collection and adipose biopsies. While this likely minimized the influence of medication on health outcomes, it remains possible that difference in medication half‐life promoted variation within our data. It is also worth noting that pre‐surgical weight loss and/or change in physical activity could have influenced the relationship between inflammation and operating time. This would seem unlikely though as there were few differences in metabolic medications, disease state or obesity classification between groups, thereby providing confidence that differences in inflammation and operating time are likely related to age. In addition, the study may be underpowered to differentiate operating time and MCP‐1 stratified in younger and older adults with or without T2D. However, co‐varying for HbA_1c_ suggests that glycaemic control does not influence the relationship between MCP‐1 and operating time, and supports the conclusion that ageing has an independent relationship to inflammation and surgery time. Lastly, it should be recognized that there was no difference in length of stay or 30 day re‐admission rates between young and old patients, thereby raising question to the clinical relevance of shorter operating time in younger adults.

In conclusion, elevated MCP‐1 is associated with higher operating time and increased metabolic risk severity in bariatric patients. While hyperglycaemia is individually and directly related to operating time, the results suggest that MCP‐1 is independently associated with increased surgical difficulty. Together, these data extend previous clinical work and demonstrate that pre‐operative inflammation is related to increased surgical risk and length of stay. Future work is necessary to address whether targeting MCP‐1 by decreasing body fat and/or attenuating inflammation pre‐operatively leads to better outcomes, particularly in older adults, as adipose tissue appears intimately involved with diabetes and cardiovascular disease.

## Conflict of Interest

No conflict of interest was declared.
